# Studying Peripheral Neuropathy and Associated Neuropathic Pain in HIV-1 Transgenic Rats: Evaluation of the Therapeutic Effect of Cannabidiol and Beta-Caryophyllene

**DOI:** 10.3390/pharmaceutics18091176

**Published:** 2026-09-17

**Authors:** Mohammed Alnoud, Jose Rios, Salique H. Shaham, Manish K. Tripathi, Khalid Benamar

**Affiliations:** 1 Institute of Neuroscience, Department of Neuro-Behavioral Health, School of Medicine, University of Texas Rio Grande Valley, Biomedical Building, McAllen, TX 78504, USAjose.rios12@utrgv.edu (J.R.); 2 Medicine and Oncology ISU, School of Medicine, University of Texas Rio Grande Valley, Biomedical Building, McAllen, TX 78504, USA

**Keywords:** HIV, peripheral neuropathy, neuropathic pain, CBD, BCP

## Abstract

**Background/Objectives**: Peripheral neuropathy affects up to 50% of people living with HIV (PLWH). Currently, no small translational rodent model accurately replicates the peripheral neuropathy observed in PLWH. The primary objectives of this study are to assess whether HIV transgenic (HIV-tg) rats develop peripheral neuropathy with pathological and pain-like behaviors similar to those observed in PLWH and to evaluate the analgesic effects of the non-psychoactive cannabis compounds cannabidiol (CBD) and β-caryophyllene (BCP) in HIV-related neuropathic pain. **Methods**: We used a comprehensive set of behavioral, pharmacological, molecular, biochemical, and proteomic approaches. **Results**: Tissue analyses showed that HIV-tg rats had significantly reduced intra-epidermal nerve fiber density and increased macrophage infiltration in the dorsal root ganglion compared with control wild-type rats. Behavioral assessments demonstrated that HIV-tg rats developed mechanical allodynia and ongoing pain. β-caryophyllene (BCP) demonstrated higher efficacy than cannabidiol (CBD) in alleviating HIV-related neuropathic pain. Proteomic analysis identified specific proteins uniquely modulated by the analgesic effects of BCP compared to vehicle in HIV-tg rats with peripheral neuropathy and neuropathic pain. **Conclusions**: The findings indicate that HIV-tg rats develop peripheral neuropathy and neuropathic pain comparable to those observed in PLWH. BCP is more effective than cannabidiol (CBD) in alleviating HIV-related neuropathic pain. Furthermore, the data suggest that BCP modulates specific proteins that may contribute to its analgesic effects in the HIV-tg model of neuropathic pain, without inducing central side effects.

## 1. Introduction

In 2024, an estimated 41 million were living with human immunodeficiency virus (HIV) [1]. Although the introduction of antiretroviral therapy (ART) has significantly extended patient survival, peripheral neuropathy continues to affect up to 50% of individuals with HIV [2]. Recent clinical data indicate an increased prevalence of HIV-associated neuropathy over the past 12 years [3]. Distal sensory polyneuropathy (HIV-DSP) is the most common clinical manifestation of HIV-associated peripheral neuropathy and remains a leading neurologic complication of HIV in the antiretroviral therapy (ART) era. Pathologically, HIV-DSP is characterized by the distal degeneration of sensory axons in a dying-back pattern [4,5,6] and loss of small unmyelinated intraepidermal nerve fibers (IENF) [5]. The high burden of neuropathy includes disability and a poor quality of life [3]. Individuals with peripheral neuropathy frequently experience neuropathic pain, which is characterized by hyperalgesia (decreased pain threshold), allodynia (increased sensitivity to touch), and persistent pain, including sensations such as pins and needles, shooting, burning, stabbing, and electric shock. Currently, no FDA-approved medications are available for HIV-DSP, and the causes of peripheral neuropathy remain poorly understood. Despite these knowledge gaps, no small translational rodent model mimics peripheral neuropathy in PLWH. Additionally, the current therapy for neuropathic pain is usually ineffective and has major side effects.

Natural products have served as sources for numerous analgesic compounds that have subsequently been developed into pharmaceuticals [7]. There is an increasing interest in exploring the therapeutic potential of cannabis. There are more than 560 components within the cannabis plant [8]. Non-psychoactive cannabinoids such as cannabidiol (CBD) have attracted considerable attention due to their potential therapeutic benefits. CBD, a minor cannabinoid present in cannabis, does not induce the adverse central nervous system effects commonly linked to cannabinoid receptor 1 (CB1).

In addition to CBD, cannabis plants contain other valuable non-psychoactive compounds, such as beta-caryophyllene (BCP) [9]. BCP, a natural bicyclic sesquiterpene, is frequently found in various essential oils. It is considered a natural selective agonist of the cannabinoid type 2 receptor (CB2) [10]. Moreover, BCP is recognized as an FDA-approved food additive [11]. It offers a range of beneficial effects, including analgesic, antidepressant, and anti-inflammatory properties [10,12,13,14,15,16,17,18,19,20].

The primary objectives of this study are (1) to determine whether HIV-tg rats develop peripheral neuropathy characterized by pathological and behavioral changes that resemble those observed in PLWH and (2) to evaluate and compare the analgesic efficacy of CBD and BCP in HIV-related neuropathic pain using the HIV-tg rat model.

## 2. Materials and Methods

All animals were tested for their pain threshold (baseline) before any treatment. Then, the CBD and BCP were administered i.p., and pain-like behaviors were assessed using von Frey, acetone, and RGS at 30, 60, 120, and 180 min post-treatment. We presented data only at 30 min, because the peak effect at this time and was maintained throughout the experiment period (3 h).

### 2.1. Animals

Male HIV-tg rats aged 6–8 months were used. The further development of the HIV-tg rats [21] particularly revolutionized the neurobiology field by moving beyond single-protein HIV studies. HIV-tg rats carry seven genes (the *gp120*, *tat*, *rev*, *vif*, *vpr*, *vpu*, and *nef*) [22]. This model has several advantages: (1) it does not require invasive induction; (2) animals continuously and constitutively express HIV-associated proteins in the absence of viral infection, similar to HIV-infected patients receiving ART, in which viral replication is substantially suppressed [23].

We purchased HIV-tg and WT rats from the University of Baltimore. Rats were group-housed (3 per cage) under a 12:12 h light–dark cycle (lights on 07:00, lights off 19:00) and provided with standard rat chow *ad libitum*. All animal care and experimental procedures used in this study were approved by the Institutional Animal Care and Use Committee (IACUC) of the University of Texas Rio Grande Valley and conducted in accordance with the National Institutes of Health accepted guidelines found in the Guide for the Care and Use of Laboratory Animals.

We conducted all experiments in a blind random manner using a validated randomization method in GraphPad 11 (quickcalcs/randomize1).

### 2.2. Drugs

The PGP 9.5, GFAP, Iba1, and CD68 antibodies were purchased from Abcam (Waltham, MA, USA). CBD and BCP were purchased from Cayman Chemical (Ann Arbor, MI, USA). Gabapentin was purchased from Sigma-Aldrich (St. Louis, MO, USA).

The vehicle consisted of dimethyl sulfoxide (DMSO), cremaphor, and saline (1:1:18).

### 2.3. The Behavioral Tests

Mechanical allodynia was assessed using the digital electronic von Frey (IITC Life Sciences, Woodland Hills, CA, USA) [24,25,26]. Rats are housed individually in plastic cages positioned on an elevated wire mesh platform and permitted to acclimate to the testing apparatus until exploratory behavior ceases. A filament is then applied with progressively increasing force, measured in grams, until the rat withdraws its paw. The apparatus automatically records the force at which this response occurs and defines it as the paw withdrawal threshold.

Cold hypersensitivity was assessed using the acetone drop method [27,28]. Animals were placed in plexiglass boxes and allowed to acclimatize. A drop of acetone was applied to the plantar surface of the paw. We recorded the time (seconds) spent elevating, licking, biting, and shaking the stimulated paw.

Heat hypersensitivity (Hargreaves test) was assessed by measuring limb withdrawal time after application of an infrared heat stimulus (Plantar test, Ugo Basile, Gemonio, Italy) [25,29]. The animals were placed in a clear plexiglass box with a dry glass floor and allowed to acclimatize. A focused infrared beam was delivered to the plantar surface of the hind paw. The paw withdrawal latency (seconds) to this stimulus was recorded.

The rat grimace scale (RGS) [30,31] is measured as an average of the five components of rodent grimacing: (1) orbital tightening or closing, (2) ear position, (3) whisker position, (4) nose bulging, and (5) cheek bulging. All five parameters measure the severity of perceived spontaneous pain. Each parameter is scored on a scale from 0 to 2, with 0 representing a normal, non-painful state, 1 indicating moderate pain, and 2 indicating severe pain.

The open field test assesses locomotion. Rats were placed in a corner of a rectangular open field test arena (40 cm length, 40 cm width, 30 cm height). A video camera was mounted on a tripod above the center of the arena, and the rats were allowed to explore for 5 min. We tracked the total distance traveled in centimeters during the 5 min exploration period from each video file and calculated it using EthovisionXT video-tracking software (Noldus Information Technology, Wageningen, The Netherlands) [32].

Body temperature was evaluated using a rectal thermometer (Physitemp; Clifton, NJ, USA) [32].

### 2.4. Immunohistochemistry (IHC)

All tissues used in the immunohistochemistry (IHC) experiments were formalin-fixed and paraffin-embedded. Skin biopsies from the hind paw area (paraffin-embedded) were cut into 5 μm sections. The sections were incubated overnight at 4 °C with the primary antibody (anti-PGP9.5 antibody) diluted 1:250. To count the number of IENFs, we used the Aspegren and Pourhamadi methods [33].

Paraffin-embedded brain and dorsal root ganglion (DRG) tissues were sectioned at a thickness of 10 μm. The primary antibodies used were Anti-Iba1 (ab178846) at 1/1000, Anti-GFAP (ab68428) at 1/250, and Anti-CD68 (ab125212) at 1/500. The secondary antibody applied was MACH 4 (Polymer) Universal HRP-Polymer (M4U534H). For immunohistochemistry (IHC) studies, 3,3′-diaminobenzidine (DAB) was used as a chromogen to visualize antibody binding, resulting in a brown precipitate at sites of antigen expression. We scanned stained sections using a Panoramic Scanner 2.2.0-MIDI (New York, NY, USA) for digital image acquisition and analyzed all images with 3DHISTECH CaseViewer software 2.4.

### 2.5. Proteomics

Frozen brain tissue from rats who had previously undergone behavioral pain assessment was ground into a fine powder under liquid nitrogen using a pre-chilled mortar and pestle for proteomic analysis, as previously described. The resulting tissue powder was transferred into 1.5 mL microcentrifuge tubes, homogenized in 400 µL of lysis buffer containing protease inhibitors, incubated on ice for 30 to 60 min, sonicated, and centrifuged at 14,000 rpm for 1 h at 4 °C. Clarified protein-containing supernatants were collected and stored at −20 °C until further processing. For proteomic analysis, 100 µg of brain protein extract, quantified by Bradford assay, was used from each sample (two experimental groups in quadruplicate). Protein extracts were prepared in lysis solution (Thermo Scientific, Waltham, MA, USA) and then reduced and alkylated for 20 to 30 min each at 95 °C for 10 min. Overnight proteolytic digestion at 37 °C was performed using a Trypsin/Lys-C enzyme mixture. Following digestion, peptides were purified using peptide cleanup columns (Thermo Scientific) and eluted in 300 µL of elution buffer. Purified peptide fractions were dried under vacuum and reconstituted in 100 µL of 0.1% formic acid. We analyzed 1 µL of each sample using an Orbitrap Exploris 240 mass spectrometer (Thermo Scientific). Peptide separation and mass spectrometric acquisition were conducted over a 120 min chromatographic gradient using a data-independent acquisition (DIA) workflow. We processed and analyzed the resulting raw mass spectrometry data using Proteome Discoverer software v. 2.5 (Thermo Scientific). We used brain tissue from the same experimental animals who underwent behavioral pain testing for both immunohistochemical (IHC) and proteomic analyses.

### 2.6. Statistics

Statistics were performed in GraphPad Prism 11 (GraphPad Software; San Diego, CA, USA), using two- and one-way ANOVA with Dunnett’s post hoc tests, *t*-test, and Mann–Whitney. Significance was set at *p* < 0.05.

## 3. Results

### 3.1. HIV-tg Rats Develop Neuropathic Pain-like Behaviors

**Figure 1:**
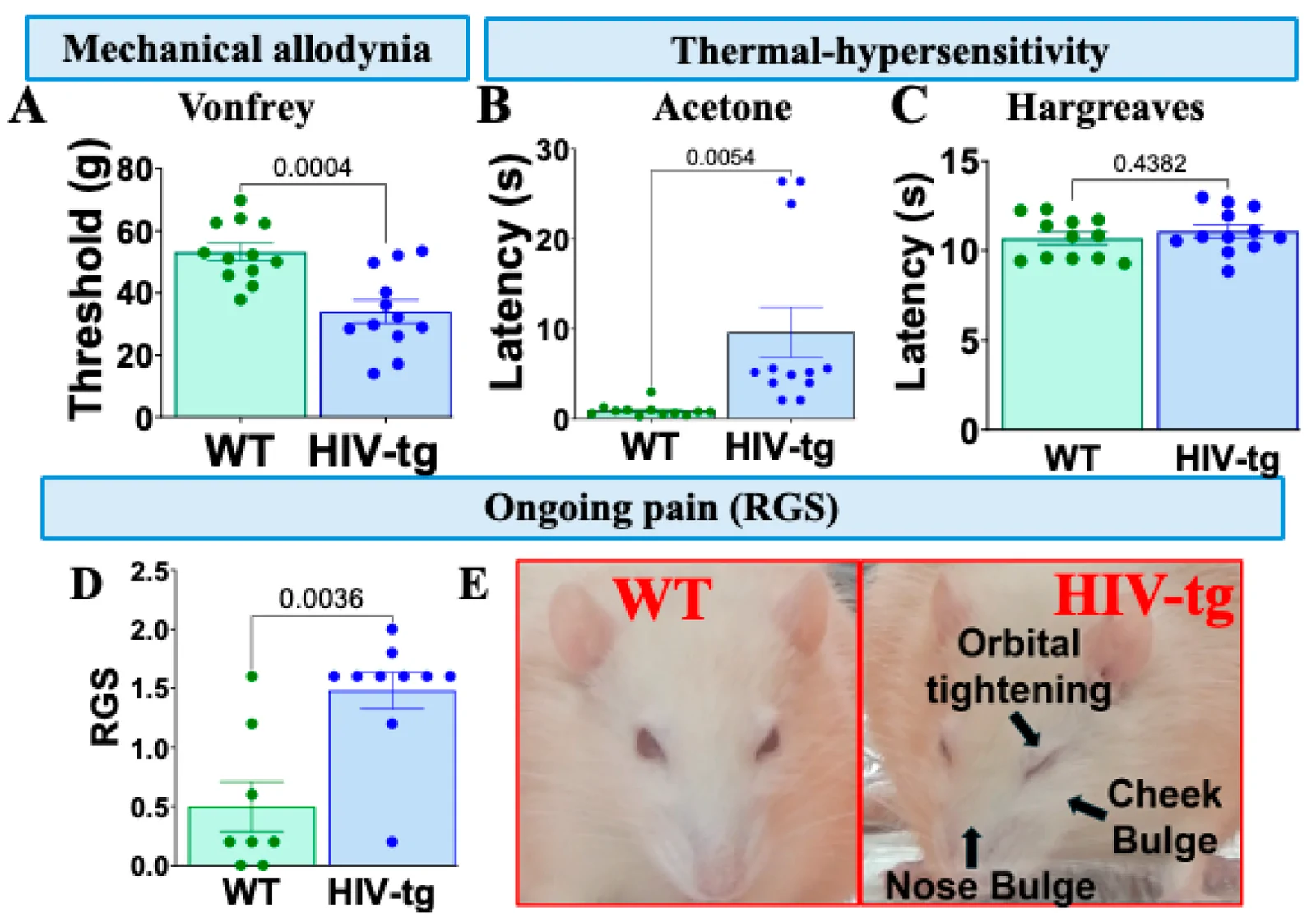
(**A**) Mechanical withdrawal thresholds of HIV-tg and WT rats using the electronic von Frey method (*n* = 12 rats per group; data are presented as mean values ± S.E.M., *p*-value from two-tailed unpaired Student’s *t*-test). (**B**) Acetone latency withdrawal of HIV-tg and WT rats (*n* = 12 rats per group; data are presented as mean values ± S.E.M., *p*-value from two-tailed unpaired Student’s *t*-test). (**C**) Heat latency withdrawal using the Hargreaves test in HIV-tg and WT male rats (*n* = 12 rats per group; data are presented as mean values ± S.E.M., *p*-value from two-tailed unpaired Student’s *t*-test). Each dot in the (**A**–**C**) represents the average of three readings. (**D**) Ongoing pain using RGS (*n* = 8–10 rats per group; data are presented as mean values ± S.E.M., *p* value from Mann–Whitney test). (**E**) Representative image of facial expression in HIV-tg rats with neuropathic pain and WT rats. RGS, Rat grimace scale; WT, wild type.

Statistical analyses indicate that HIV-tg rats develop significantly higher mechanical allodynia compared to wild-type (WT) controls (Figure 1A, *t*-test). HIV-tg rats also exhibit significantly increased cold hypersensitivity (Figure 1B, *t*-test), whereas no significant difference in heat hypersensitivity is observed (Figure 1C, *t*-test). Additionally, HIV-tg rats show higher RGS scores than WT controls (Figure 1D, Mann–Whitney test). Figure 1E presents representative facial expressions in HIV-tg and WT rats. The HIV-tg rats demonstrate the following features: (1) tightly closed eyelids, (2) ears rotated outward and curled inward, (3) nose and cheeks flattened with an elongated nose, and (4) whiskers straightened, bunched together, and positioned horizontally to the cheeks.

### 3.2. HIV-tg Rats Develop Neuroinflammation

**Figure 2:**
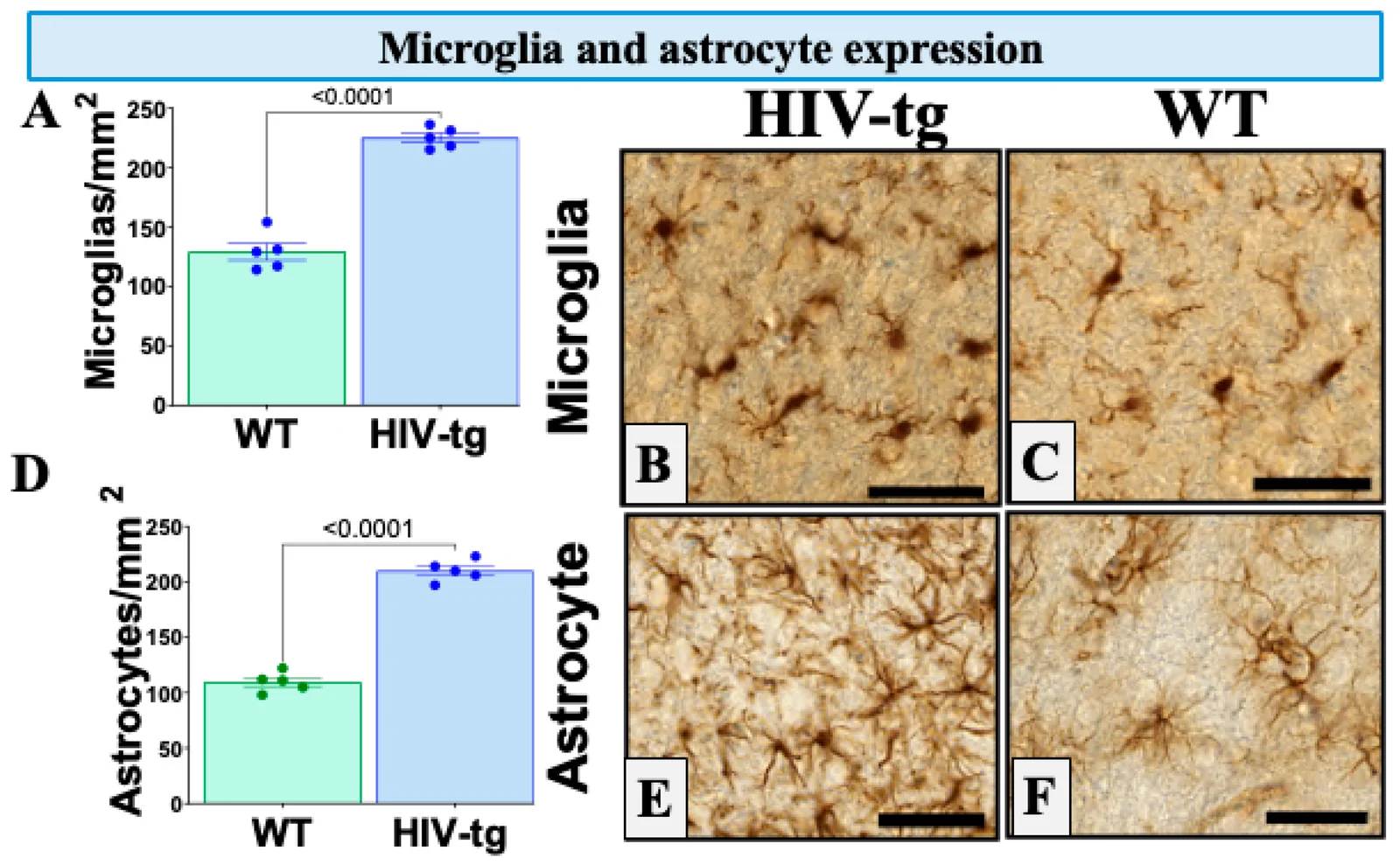
Expression of Iba1 (**A**) and GFAP (**D**) in HIV-tg rats with neuropathic pain and wild-type (WT) controls. Data are presented as means ± S.E.M., *n* = 5 rats per group. Scale bar = 0.05 mm. Representative images show increased Iba1-positive microglia in HIV-tg (**B**) compared to WT (**C**) and increased GFAP-positive astrocytes in HIV-tg (**E**) compared to WT (**F**). These images were captured from the periaqueductal gray, a key brain region involved in the descending pain modulatory system.

To validate HIV-tg-related neuropathic pain, we next assessed neuroinflammation that plays a key role in neuropathic pain [34,35,36,37]. We examined microglial and astrocytic levels in HIV-tg and WT rats. HIV-tg showed significantly increased expression of the microglial marker Iba1 (Figure 2A) and the astrocyte marker GFAP (Figure 2D), demonstrating neuroinflammation in HIV-tg compared with WT. Representative images of microglial Iba1 immunostaining in HIV-tg (B) and WT (C) are shown, as are representative images of astrocytes and GFAP immunostaining in HIV-tg (E) and WT (F).

### 3.3. HIV-tg Rats Develop Peripheral Neuropathy

**Figure 3:**
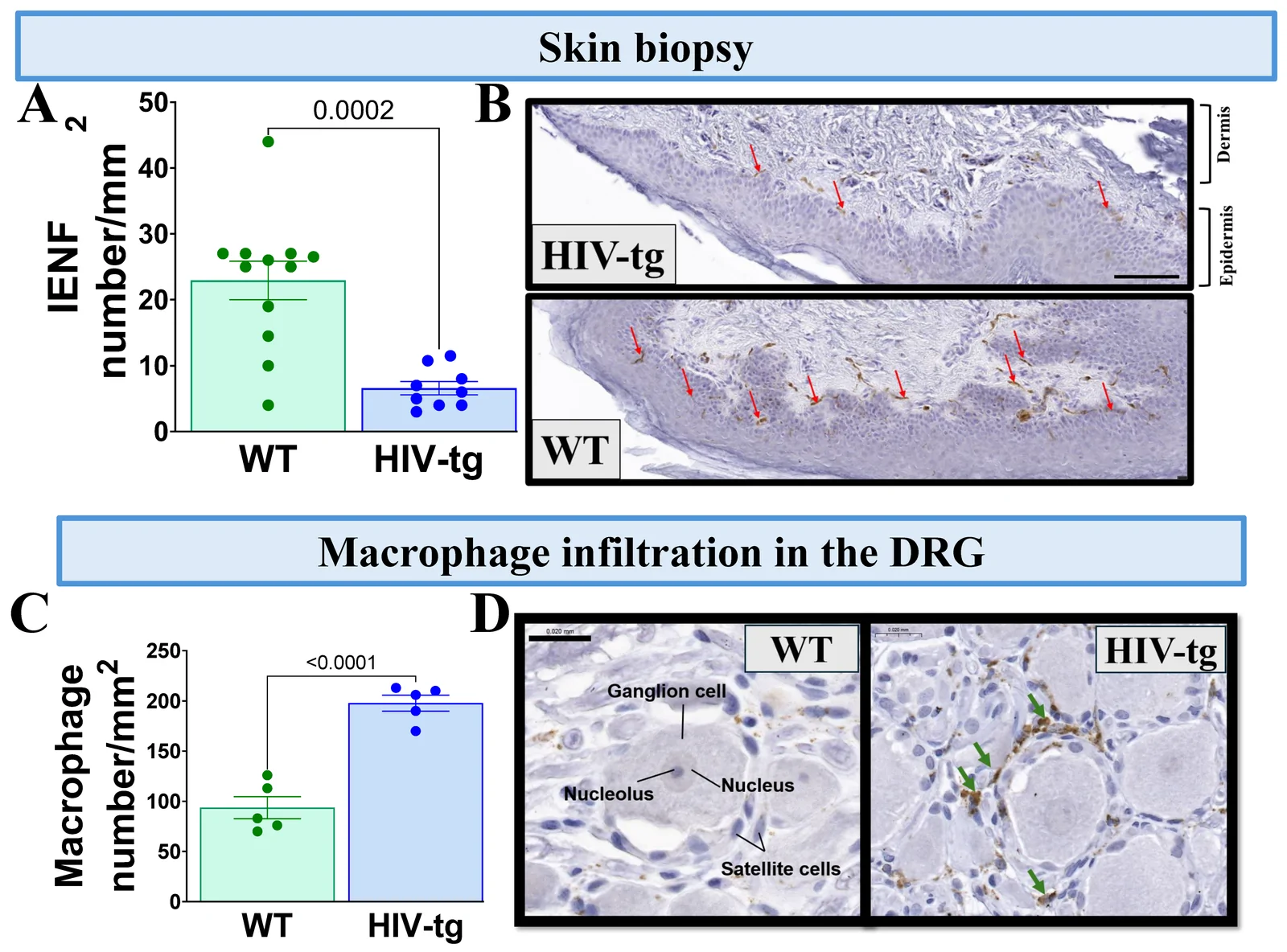
(**A**) Quantification of IENF of HIV-tg and WT rats (*n* = 9–12 rats per group; data are presented as mean values ± S.E.M., *p*-value from Mann–Whitney test). (**B**) Representative PGP9.5 staining of hind paw skin of HIV-tg and WT rats (scale bars, 50 μm). Red arrows show PGP9.5 staining. (**C**) Quantification of macrophage infiltration into DRG of HIV-tg and WT rats (*n* = 5 rats per group; data are presented as mean values ± S.E.M., *p*-value from two-tailed unpaired Student’s *t*-test). (**D**) Representative DRG staining of CD68 of HIV-tg and WT rats (scale bars, 20 μm). Green arrows show CD68 staining.

We quantified the number of IENFs using immunostaining for the neuronal marker protein gene product 9.5 (PGP 9.5). Histopathological data from skin biopsies from the plantar area of the hind paw show a significant decrease in IENF number in HIV-tg compared to WT rats. (Figure 3A, Mann–Whitney test). An image representative of the quantification of IENF density using immunohistochemistry with PGP 9.5 antibody showed a reduced number of IENF in HIV-tg compared to WT (Figure 3B).

We also evaluated macrophage infiltration into the dorsal root ganglion (DRG). We used CD68, a macrophage marker. The number of CD68-positive cells was significantly increased in the L4–5 DRG of HIV-tg compared to WT rats (Figure 3C, *t*-test). Representative images of CD68 staining in L4–5 DRG from HIV-tg and WT rats are also shown (Figure 3D).

### 3.4. CBD and BCP Administration Resulted in a Reduction in Neuropathic Pain-like Behaviors in HIV-tg Rats

**Figure 4:**
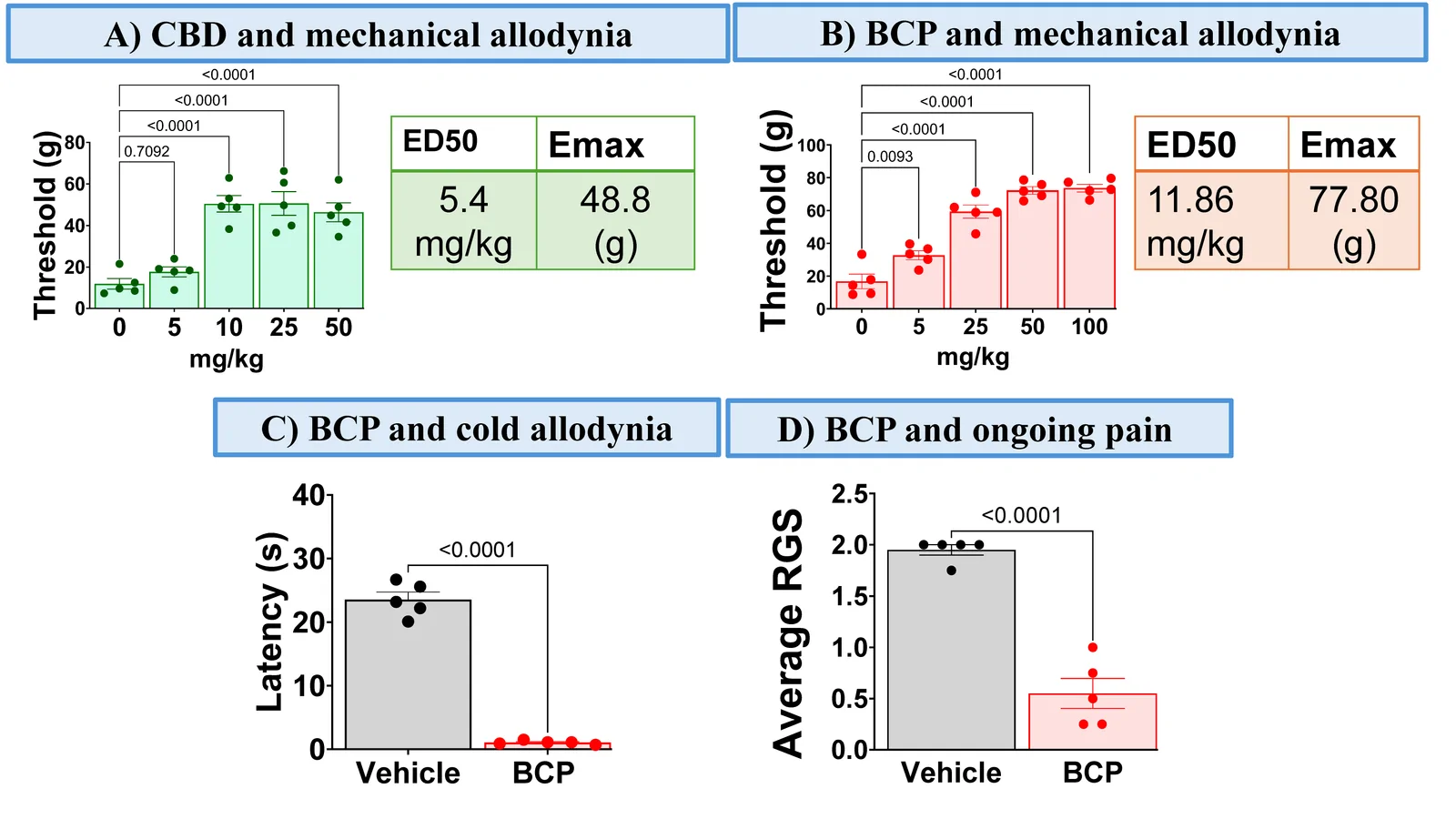
The effects of i.p. administration of CBD (**A**) and BCP (**B**) on mechanical allodynia in HIV-tg rats (*n* = 5 per group) are shown as mean values ± S.E.M., with *p* values determined by one-way ANOVA and Dunnett’s test. The effects of BCP on thermal hypersensitivity (**C**) and ongoing pain (**D**) in HIV-tg rats (*n* = 5 per group) are also presented as mean ± S.E.M., with *p* values from a two-tailed unpaired Student’s *t*-test. ED_50_ refers to the median effective dose, and Emax indicates the maximal effect. ED_50_ values were determined by nonlinear regression using GraphPad Prism (version 11; GraphPad Software, Inc., La Jolla, CA, USA).

Intraperitoneal (i.p.) administration of CBD at doses ranging from 5 to 50 mg/kg produced a dose-dependent (D-R) reduction in mechanical allodynia, as demonstrated in Figure 4A (one-way ANOVA with Dunnett’s post hoc test), with an ED_50_ of 5.4 mg/kg and an Emax of 48.8 g.

Similarly, BCP administered at 5 to 100 mg/kg (i.p.) also resulted in a dose-dependent reduction in mechanical allodynia, as shown in Figure 4B (one-way ANOVA with Dunnett’s post hoc test), with an ED_50_ of 11.86 mg/kg and an Emax of 77.80 g. The D-R analysis indicates that BCP exhibits a higher Emax than CBD, suggesting higher efficacy. However, CBD is more potent because its ED_50_ is lower than BCP’s. Given that BCP showed higher analgesic effects compared to CBD, we tested the most effective dose of BCP for cold allodynia and ongoing pain. BCP showed a strong significant effect in attenuating thermal hypersensitivity in the acetone test (Figure 4C, *t*-test) and significantly reduced RGS (Figure 4D, *t*-test).

**Figure 5:**
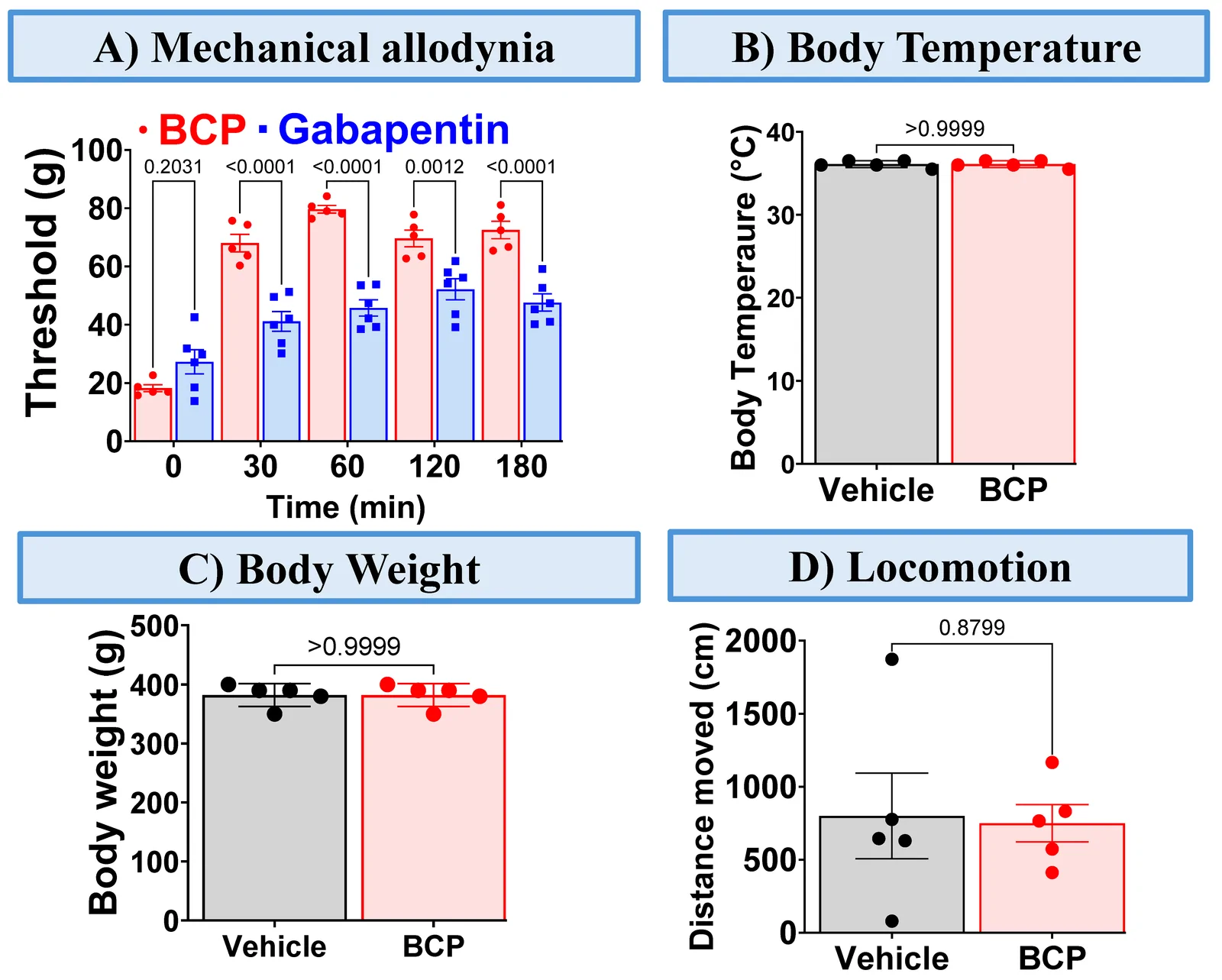
Comparison of the analgesic effect of BCP and gabapentin (**A**) in HIV-tg rats with neuropathic pain (*n* = 5 rats per group; data are presented as mean values ± S.E.M., *p*-value from two-way ANOVA with Bonferroni’s multiple comparisons). The lack of adverse effects of BCP on body temperature (**B**), body weight (**C**) and locomotion (**D**) (*n* = 5 rats per group, data are presented as mean values ± S.E.M. *p* value from two-tailed unpaired Student’s *t*-test).

In our subsequent comparison, we evaluated the analgesic effects of BCP alongside those of the positive control, gabapentin (Figure 5A). The results were particularly intriguing. BCP showed a superior analgesic effect compared to gabapentin (Figure 5A). The dose (100 mg/kg) and route (i.p.) of gabapentin we used are within the range of doses that show an analgesic effect in several neuropathic pain models in rats with consistent results [38,39,40,41].

BCP was also evaluated for its impact on body temperature, locomotor activity, and body weight. The data show no significant adverse effects on body temperature (Figure 5B), body weight (Figure 5C), and the open field test (Figure 5D) in HIV-tg rats compared to the vehicle.

### 3.5. Proteomics

**Figure 6:**
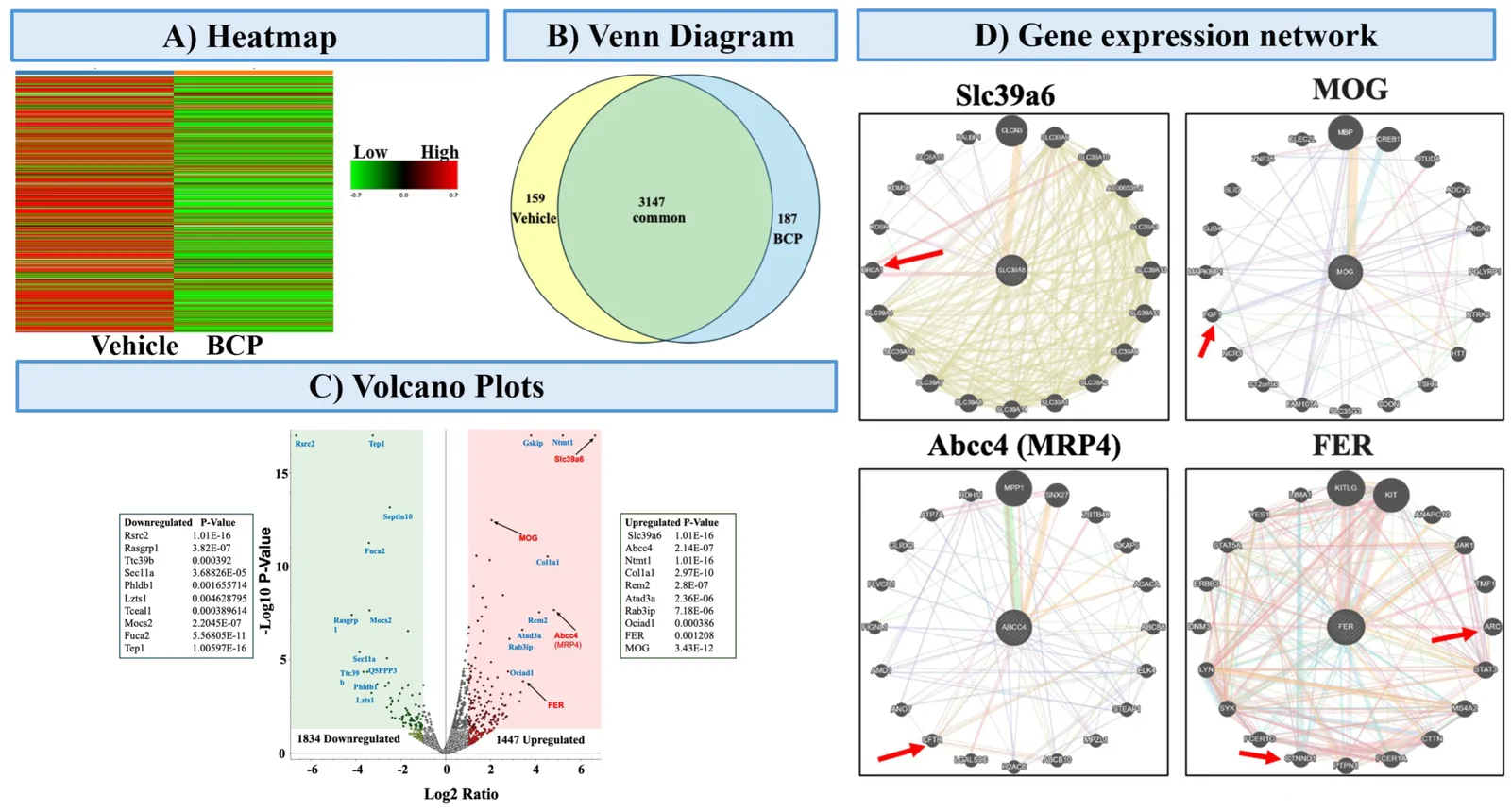
(**A**) Heat map of grouped samples from *n* = 4 vehicle- and *n* = 4 BCP-treated rat brains. Rows represent individual proteins, and columns correspond to analyzed samples. Proteins significantly decreased are shown in green, while those significantly increased are shown in red. Color intensity reflects the magnitude of difference relative to the average value. (**B**) The Venn diagram illustrates unique and shared brain proteins between the HIV Tg (vehicle control) and BCP treatment groups. A total of 3306 proteins were identified in HIV Tg, with 3334 proteins in BCP treatment. Of these, 3147 proteins were common to both groups, with 159 unique to the control and 187 unique to the BCP treatment group (*n* = 4 rats per group). (**C**) Volcano plot summarizing significantly upregulated and downregulated proteins. The top 10 upregulated and top 10 downregulated differentially expressed proteins are indicated. Black arrows denote genes involved in pain. (**D**) Network diagrams display GeneMANIA analysis of genes upregulated in BCP-treated tissue compared to control. Each circular plot depicts gene–gene interaction networks for pain-related genes. Nodes represent genes, with larger nodes indicating higher connectivity or importance within the network. Edges represent functional associations between genes, with different colors indicating interaction types such as co-expression, physical interaction, shared protein domains, genetic interaction, and predicted pathways. The red arrow indicates genes involved in the pain reduction pathway.

The clustering heatmap (Figure 6A) presents the expression profiles of differentially expressed brain proteins in HIV-tg rats with peripheral neuropathy and neuropathic pain treated with vehicle or BCP. Distinct clustering patterns reveal treatment-specific proteomic signatures. Each row represents a unique protein, and each column corresponds to a treatment group, with four replicates per group. The heatmap uses a color gradient to indicate protein abundance, with red denoting high abundance and green indicating low abundance.

The Venn diagram (Figure 6B) illustrates the overlap and distinctions among proteins identified in HIV-tg rats with peripheral neuropathy and neuropathic pain treated with the vehicle or BCP. Specifically, 159 proteins were unique to the vehicle group, while 187 were unique to the BCP group. A total of 3147 proteins were common to both groups.

The volcano plot (Figure 6C) depicts the distribution of proteins significantly upregulated or downregulated in HIV-tg rats with peripheral neuropathy and neuropathic pain treated with BCP compared with the vehicle group. In the BCP group, 1447 proteins were upregulated relative to the control. Among the top ten upregulated proteins, four were associated with pain: Slc39a6, MOG, Abcc4, and FER. Based on our literature search, none of the top ten downregulated proteins were associated with pain.

Figure 6D presents a network analysis, conducted using the GeneMANIA database, of four genes upregulated by BCP treatment in HIV-tg rats with neuropathic pain. The analysis indicates that Slc39a6 is associated with BRCA1, which is indirectly linked to pain relief through RARβ agonist activity [42]. MOG is connected to FGF1, a factor known to reduce inflammation in neuropathic pain [43]. Abcc4 is associated with CFTR, which suppresses downstream proinflammatory NFκB signaling [44], a pathway responsible for inflammation and pain. FER is linked to ARC and CTNND1; ARC mediates pain relief by decreasing AMPAR transcription [45], and CTNND1 encodes P120-catenin, a negative regulator of the NFκB neuroinflammatory signaling pathway [46].

## 4. Discussion

This study reveals two important findings. (1) HIV-tg rats develop peripheral neuropathy and neuropathic pain-like behavior similar to those observed in PLWH. (2) BCP has superior analgesic effects in HIV-tg rats with neuropathic pain compared to CBD and gabapentin without CB1 central adverse effects.

ART treatment has successfully reduced the prevalence of AIDS, and now the disease is primarily managed as a chronic disease [47]. However, a new spectrum of problems has emerged because of the prolonged presence of HIV viral proteins. It appears that the prolonged toxic actions of HIV proteins play a role in the neuropathogenesis of HIV disease in the ART era [48,49,50,51,52,53] rather than the virus by itself. Therefore, the use of HIV-tg rats that carry HIV-related proteins (gp120, tat, rev, vif, vpr, vpu, and nef) [22] seems suitable for studying neuropathy and neuropathic pain in the context of HIV.

HIV-associated peripheral neuropathy is among the most common neurologic complications in individuals with HIV during the ART era. We conducted histopathological studies to assess peripheral neuropathy in HIV-tg rats. The IENF density is a key clinical diagnostic tool for peripheral neuropathy, including HIV-associated sensory neuropathy [4,54,55]. Skin biopsy data from the plantar area of the hind paw show a significant decrease in IENF number in HIV-tg rats with neuropathic pain compared to WT rats. Our data provided the first scientific evidence for peripheral nerve damage in HIV-tg rats.

We also assessed whether macrophages infiltrate the DRG. Macrophage infiltration into DRG is a marker of PNS injury [56,57], and it has been shown to occur in HIV patients with DSP [58]. Our data show a significant increase in macrophage infiltration into DRGs in HIV-tg rats with neuropathic pain compared to WT rats.

To validate HIV-tg-related neuropathic pain, we assessed neuroinflammation, as inflammation plays a central role in both the development and persistence of neuropathic pain [34,35,36,37]. Inflammation not only serves as a driving force for neuropathic pain but is also found in HIV individuals [59], including HIV individuals on suppressive ART [60,61]. Our findings show that in HIV-tg rats, Iba1, a well-known microglial marker, and GFAP, a well-known astrocytic marker, are significantly increased in the brain.

Taken together, these data support peripheral neuropathy in HIV-tg rats and the presence of neuroinflammation, a key characteristic of chronic pain.

Given that neuropathic pain is mainly caused by nerve damage from peripheral neuropathy, we tested HIV-tg rats for neuropathic pain. Our data show that HIV-tg rats developed sensory pain in line with previous studies [62]. Moreover, we showed that HIV-tg also develops ongoing pain. These data indicate that HIV-tg rats develop a neuropathic pain-like condition with behavioral features (such as sensory and ongoing pain) that mimic neuropathic pain in PLWH.

Next, we use this model to explore the potential use of cannabis components to reduce HIV-related neuropathic pain. This issue is significant because current pharmacological treatments for neuropathic pain management, including gabapentin, which is considered the gold standard, often demonstrate limited effectiveness and are associated with various side effects [63,64,65].

We investigated the effects of two major non-psychoactive cannabis compounds: CBD and BCP.

These compounds were specifically selected for their therapeutic potential without the psychoactive effects commonly associated with cannabis.

The administration of CBD led to a significant D-R reduction in mechanical allodynia in HIV-tg rats. Similarly, BCP decreases mechanical allodynia in a D-R manner. Analysis of the D-R profile shows that BCP has a notably higher Emax than CBD, indicating that BCP is more effective at mitigating mechanical allodynia.

We also evaluated BCP’s analgesic efficacy for thermal hypersensitivity and ongoing pain. The outcomes were promising, indicating that BCP significantly decreased thermal hypersensitivity in the acetone test. Additionally, BCP treatment notably reduced RGS, demonstrating its effectiveness in controlling ongoing pain. This is especially important because clinical trials of pain-relief drugs often focus on behavioral outcomes driven by mechanical or thermal stimuli, which do not fully reflect the chronic pain relevant to human treatment. By evaluating the effectiveness of BCP against ongoing pain, we obtain a valid and reliable assessment that more accurately predicts its use in managing HIV-related neuropathic pain in humans.

We also evaluated the analgesic effects of BCP alongside the positive control, gabapentin. The results were particularly intriguing, as BCP showed a significantly stronger antiallodynic effect than gabapentin, which is widely recognized as the gold standard treatment for neuropathic pain.

We also tested BCP for potential CB1 central side effects. We found that BCP does not affect body temperature, body weight, or locomotion.

A proteomics approach was employed to elucidate the mechanisms underlying the enhanced analgesic effect of BCP in HIV-tg rats with neuropathic pain. Proteomic analysis demonstrated that, although BCP shares some molecular targets with the vehicle, it also modulates a distinct set of proteins. In the BCP-treated group, 1447 proteins were upregulated compared to the control group. Among the top ten proteins upregulated by BCP in HIV-tg rats with neuropathic pain, four proteins—Slc39a6, MOG, Abcc4, and FER—are associated with pain pathways and may play a significant role in mediating the analgesic effect of BCP in HIV-tg-related neuropathic pain.

In summary, these data show that HIV-tg rats develop a neuropathic pain-like condition with behavioral features (such as sensory and ongoing pain) and pathological changes (such as reduced IENF) that resemble neuropathic pain and peripheral neuropathy in PLWH. These findings indicate that HIV-tg rats represent a suitable translational model for studying HIV-related neuropathic pain and peripheral neuropathy. Additionally, our findings demonstrate that BCP has superior antiallodynic effects compared to CBD and gabapentin. These findings suggest that tBCP may offer a more effective alternative for pain relief compared to gabapentin, highlighting its potential as a valuable option in pain management. Proteomic analyses further demonstrate that BCP modulates specific proteins that may underlie its analgesic effects in HIV-tg models of neuropathic pain, without inducing central side effects.

A limitation of our studies is that we did not address the biological variable of sex. Although proteomic analysis identified key proteins affected by BCP’s analgesic effect, further studies are needed to validate the functional significance of the upregulated proteins in HIV-tg rats. We used the entire brain for proteomic analysis and acknowledge the lack of regional resolution as a limitation. Finally, although the genes identified in this study require direct experimental validation, the findings offer independent biological support for the potential relevance of several candidate genes and their associated molecular networks identified through unbiased proteomic analysis.

## Data Availability

The data supporting the findings of this study are available from the corresponding author upon request.
